# The value of utility payment history in predicting first-time homelessness

**DOI:** 10.1371/journal.pone.0292305

**Published:** 2023-10-09

**Authors:** Colin D. Middleton, Kim Boynton, David Lewis, Andrew M. Oster

**Affiliations:** 1 Department of Mathematics, Eastern Washington University, Cheney, Washington, United States of America; 2 Avista Utilities, Spokane, Washington, United States of America; 3 Homeless Management Information System, City of Spokane, Spokane, Washington, United States of America; University of Medical Sciences, Ondo City, NIGERIA

## Abstract

Homelessness is a costly and traumatic condition that affects hundreds of thousands of people each year in the U.S. alone. Most homeless programs focus on assisting people experiencing homelessness, but research has shown that predicting and preventing homelessness can be a more cost-effective solution. Of the few studies focused on predicting homelessness, most focus on people already seeking assistance; however, these methods necessarily cannot identify those not actively seeking assistance. Providing aid before conditions become dire may better prevent homelessness. Few methods exist to predict homelessness on the general population, and these methods use health and criminal history information, much of which may not be available or timely. We hypothesize that recent financial health information based on utility payment history is useful in predicting homelessness. In particular, we demonstrate the value of utility customer billing records to predict homelessness using logistic regression models based on this data. The performance of these models is comparable to other studies, suggesting such an approach could be productionalized due to the ubiquity and timeliness of this type of data. Our results suggest that utility billing records would have value for screening a broad section of the general population to identify those at risk of homelessness.

## Introduction

The United States Department of Housing and Urban Development (HUD) defines categories of homelessness with their *“Category 1: Literally Homeless”* as

1) Individual or family who lacks a fixed, regular, and adequate nighttime residence, meaning:
(i) Has a primary nighttime residence that is a public or private place not meant for human habitation;
(ii) Is living in a publicly or privately operated shelter designated to provide temporary living arrangements (including congregate shelters, transitional housing, and hotels and motels paid for by charitable organizations or by federal, state and local government programs); or
(iii) Is exiting an institution where (s)he has resided for 90 days or less and who resided in an emergency shelter or place not meant for human habitation immediately before entering that institution [1]

The agencies that collected the data utilized in this work used HUD’s *“Category 1: Literally Homeless”* definition of homeless. To be consistent with the data, we adopt the same definition.

**Fig 1:**
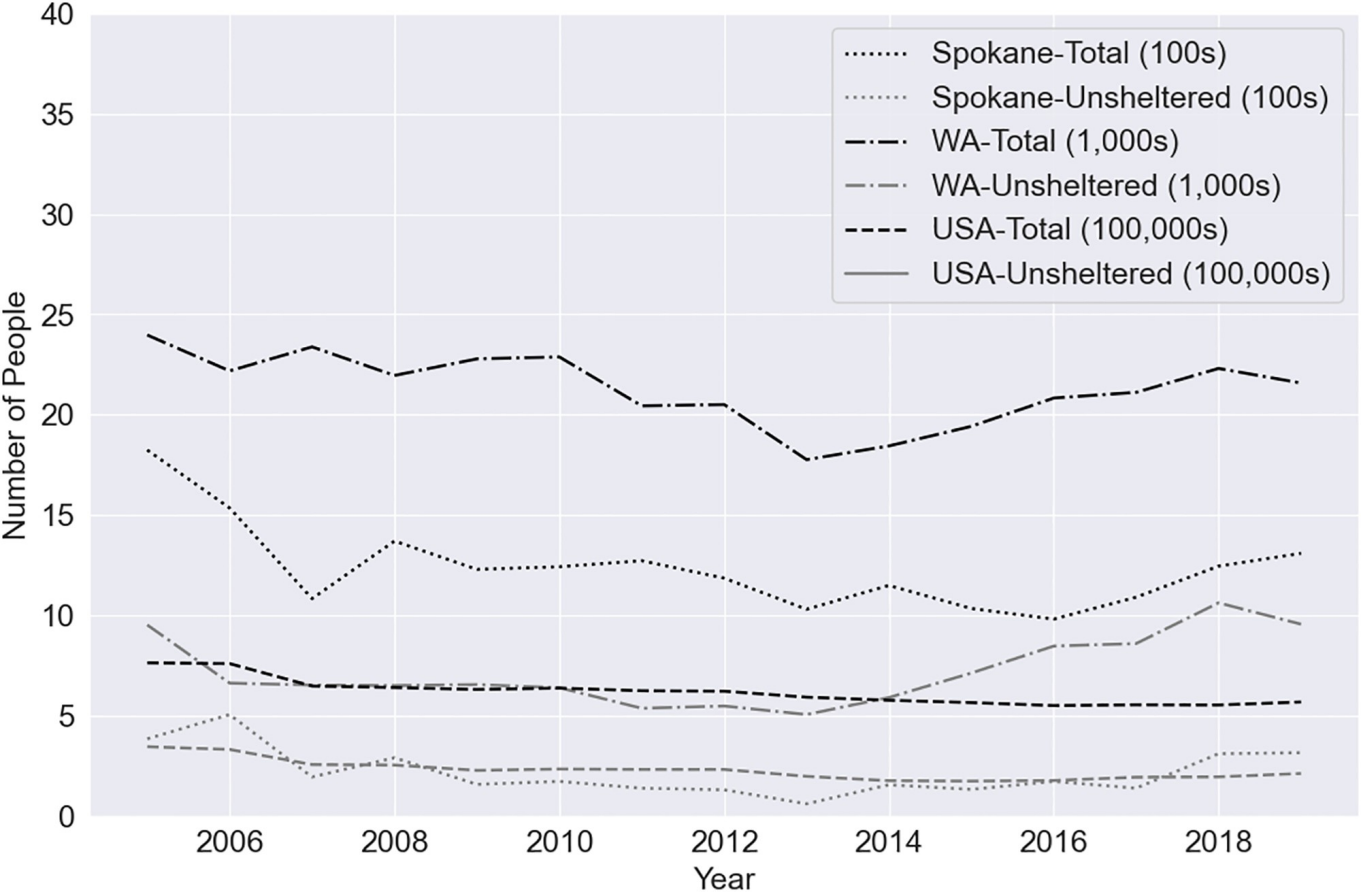
Annual homelessness counts. The counts for Spokane County (Spokane) are displayed in 100s, the counts for Washington State (WA) are displayed in 1000s, and the counts for the United States of America (USA) are displayed in 100,000s [2].

Homelessness in the United States persists despite the efforts of various homelessness assistance programs. There has been a decrease of 42% in homelessness in the U.S. from 2005 until 2020 according to the Department of Housing and Urban Development (HUD), but over 442,000 people were still recorded as experiencing homelessness in 2020 through HUD’s Point in Time (PIT) count program [2]. In Washington State, homelessness has decreased by 28% from 2005 to 2020, but 17,264 people were still reported experiencing homelessness in 2020 [2]. In this work, we consider homelessness in Spokane County, Washington, USA. Spokane County has seen homelessness levels decrease by 32% from 2005 to 2020 with 1,244 people recorded as experiencing homelessness in 2020. These numbers include individuals sheltered in emergency shelters, sheltered in transitional housing, and unsheltered [2]. The rates of homelessness at the national, state, and county level are displayed in Fig 1. Since the data collection period of this study (2015–2020), homelessness has further decreased at the national level (397,660 in 2022), but increased at the state (17,748 in 2022) and county (1,413 in 2022) levels [2].

Programs focused on providing assistance to people experiencing homelessness are having an impact. HUD’s January 2019 survey found that “there were 144,000 more permanent supportive housing (PSH) beds dedicated to people with chronic patterns of homelessness than there were in 2007 (a 380% increase)” [3]. Likely because of this increase in resources, the rate of chronic homelessness (people who have experienced homelessness for at least 12 months in the last three years) has declined by 20% from 2007 to 2019 in the U.S. [3]. Current assistance programs and the positive impacts they have on people in need represent a significant accomplishment, but these programs largely focus on providing support to people already experiencing homelessness. Prevention programs are needed to substantially reduce homelessness into the future.

Homelessness prevention programs (HPPs) focus on preventing people from experiencing homelessness. These programs include permanent deep rental housing subsidies, eviction prevention programs, community based services such as short term financial assistance, education, and job placement. These are counterpoint to the more ubiquitous, homeless assistance programs (HAPs), which assist people who currently experiencing homelessness, such as shelters and other emergency services. One key benefit of an HPP compared to an HAP is that it spares people the trauma typical of experiencing homelessness. Another key attribute of HPPs compared to HAPs is their reduced cost. The services that HPPs typically offer are less expensive for taxpayers than those provided by HAPs [4]. Research has shown that many types of HPPs are cost-effective in practice, meaning that treating the socioeconomic issue of homelessness with HPPs instead of HAPs is at least as effective at keeping people in stable housing and requires less expenditure than HAPs [4–7].

A study analyzing several types of HPPs found several to be effective at preventing homelessness and some to be cost-effective, though research in this area is limited. HPPs can employ a variety of interventions including shelter diversion programs, permanent shallow rent subsidies, targeted interventions and transition planning for vulnerable populations [4]. A permanent deep rental housing subsidy program, one of the most promising types of HPP, had “67 percent of families who successfully used their [rent subsidy] voucher to lease housing, homelessness was prevented entirely” [4]. A New York based HPP called HomeBase offering a variety of services was estimated to have saved taxpayers $140 per family served [4, 5]. Critical Time Intervention, a more established program aimed at preventing psychiatric patients from experiencing homelessness, significantly reduced the likelihood a patient experiences homelessness [4]. An eviction prevention program in Chicago that distributed rent assistance based on at-risk individuals calling a Homelessness Prevention Call Center observed that those awarded assistance were 76% less likely to enter a shelter than those that were not awarded funding and the authors claim the program’s efficiency would likely be higher with better targeting strategy to identify those most at risk [6]. Using the Homelessness Prevention Call Center mentioned previously, Evans et al. estimated that the costs of assisting someone were $10,300 while the benefits were greater than $20,000, a net savings of $9,700 per person, and postulated that the costs of assisting people could be much reduced with improved targeting [6]. Homeless prevention can also be effective on a larger scale. In 2009, the United States government distributed $1.5 billion through the Homeless Prevention and Rapid Rehousing Program which promoted HPPs. According to the National Alliance to End Homelessness this support of HPPs largely drove the 1% decline in homelessness between 2009 and 2011, which is notable considering the economic recession during that time [7].

There is an important hypothesized pattern in the pathology of homelessness that is likely the source of HPPs’ effectiveness—a gradual and measurable divergence from financial stability. We hypothesize that individuals that eventually experience homelessness begin their journey as indistinguishable from the general population, but, as time progresses, these individuals begin to show signs of financial distress and become more and more distinct from the financially healthy population (and more identifiable as at-risk) as they progress towards experiencing homelessness. As this progression continues, and especially once an individual experiences an episode of homelessness, it becomes more difficult to change their financial trajectory and help them reestablish their financial independence. This hypothesis is supported by Shinn et al., who found that “the single best predictor of eventual homelessness is having previously been in a shelter,” indicating that once someone experiences homelessness, it is much more difficult for them to regain financial stability [4]. Moreover, the longer one experiences homelessness, the more difficult and expensive it is to stably house them [8]. The benefit of HPPs is that they endeavor to change the trajectories of at-risk individuals early in their progression towards homelessness. If an intervention is performed earlier, a smaller, less expensive course correction is necessary.

Identifying individuals to enroll in HPPs is a difficult task because everyone is a candidate as anyone might experience homelessness at some point. This is why it is important to predict homelessness at the population level. The path to homelessness varies across individuals; some may persist in a financially stressed state without ever experiencing an episode of homelessness, while others may be financially stable until a large unforeseen expense is incurred [9]. We theorize that the majority of people who experience homelessness exist in an identifiable state of financial distress for a period of time prior to the episode. Because of the ability for some individuals to maintain a state of financial instability but never experience homelessness, even the best prediction systems will likely produce false positives. One concern with HPPs is whether there are portions of the population that would not benefit from assistance. However, a study on New York’s HomeBase program in 2013 found that even those predicted most likely to experience homelessness still benefit from services [7]. This finding provides statistical backing to the notion that no one is beyond help.

Currently, screening for HPPs is almost entirely performed by healthcare workers, who evaluate each case and determine the level of support needed. High volumes of individuals seeking assistance, especially in large cities, can slow down this system immensely. The use of statistical models as a screening method shows promise in facilitating identification of those in need of aid by increasing processing speed and prediction accuracy [4, 7]. One study found that switching from humans to a statistical model for applicant screening reduced the number of false negatives from 28.4% to 8.1% [4], while another study found that a statistical approach as well as a point system approach both outperformed worker decisions by achieving higher hit rates with lower false positives [7]. Similarly, Greer et al. found that statistical models as screeners are more efficient than human healthcare workers [10].

Most previous research in predicting homelessness has focused on populations already identified as at risk or were self selected due to seeking some form of government assistance [7, 8, 11–14]. In [7, 8, 11–14], the methodologies employed included logistic regression, K-means clustering, Cox survival analysis, and machine/deep learning approaches. One major cause for the dearth of research on predicting homelessness for the general population is the difficulty in attaining data that covers the entire population, are relevant to predicting homelessness, and are timely enough to be useful in the production setting of an HPP.

The single population level study available at the time of writing was published by Byrne et al. in 2020. This study predicted homelessness for “5,050,639 individuals aged 11 years and older who were included in a linked dataset of administrative records from multiple state-maintained databases in Massachusetts for the period from 2011–2015,” which covers “more than 98% of Massachusetts residents” [15]. Their study used a logistic regression model that was trained on predictors related to: mental health, substance abuse, emergency medical service use, incarceration, veteran status, and demographics. Their model achieved admirable performance with a True Negative Rate of 0.954, a True Positive Rate of 0.778, a Balanced Accuracy of 0.864, and an Area Under the Receiver-Operator Curve of 0.94 [15]. The database used by Byrne et al. was the “All-Payer Claims Database (APCD), which aggregates health care claims from all public and private payers” [15]. This database clearly contains valuable information for predicting homelessness, but it has limitations. At the time of writing, only “18 States have legislation mandating the creation and use of APCDs or are actively establishing APCDs,” though “more than 30 States maintain, are developing, or have a strong interest in developing an APCD” [16]. The main limitation of healthcare claims data is its timeliness. Medical claims take time to process and work their way through the healthcare and insurance systems, causing a lag between when the event that caused the claim occurs and when the claim ends up in the database. On top of this time lag is the additional time period between when the event occurs and when the claim is filed. Medicare will accept a claim up to “12 months (or 1 full calendar year) after the date when the services were provided” [17]. Since the goal of prediction and prevention is to intervene early, this data source has severe limitations for production system.

Previous studies have found relevant data for predicting homelessness, but there is a gap in the research for data sources that are both timely and accessible. The primary goal of this paper is to demonstrate the value of customer utility billing data in the prediction of first-time homelessness. These data are widely available and are updated monthly, thus, predictive models using this framework would allow HPPs to identify at risk individuals for assistance and have time to assist them before they experienced homelessness. A data source with these three important qualities (relevance, timeliness, and accessibility) has yet been presented in the field of homelessness prediction. In particular, we demonstrate the usefulness of this data by training interpretable logistic regression models and evaluating their performances. A secondary outcome of this project is a widely usable, population level, initial screening technique based solely on utility billing records. Individuals identified as being at risk of experiencing homelessness could be contacted and further information gathered from them to perform further screening steps. The remainder of the paper is constituted by: the materials and methods, results, discussion, and conclusion. The materials and methods are organized into subsections addressing data preparation and pre-processing, model description, and model evaluation methods. The results describe the performance of the model. Finally, the discussion provides context for these results, and the conclusion summarizes the main points of the paper and suggests future directions of inquiry.

## Materials and methods

We used data obtained from Avista Utilities and the City of Spokane in Washington state. Avista Utilities provided monthly residential utility customer billing information for electricity and natural gas utilities as well as information related to collections activity. The City of Spokane provided monthly combined water, sewer, and garbage utility billing information, in addition to instances of homelessness recorded in their Community Management Information System (CMIS), which complies with HUD’s Homeless Management Information System regulations.

Utility billing data is attractive for two key reasons: 1) it can be used as a proxy for account holder’s financial health and 2) its ubiquity. Utility billing data contains information related to individuals’ financial health such as amount owed to the utility company and number of times a bill payment was missed. Risk of experiencing homelessness is hypothesized to be closely linked with an individual’s financial health. In addition, utility billing data is ubiquitous as almost all households pay some form of utility bill, making this data available for at least one member of most households across the United States and likely in most capitalist economies. This data may exist in different forms and in different agencies, but similar information on amounts owed and payment default likely exist in all residential utility-customer situations.

For convenience, we refer to a person-location-month within twelve months, or one year, of being recorded experiencing first-time homelessness as a “positive case,” or simply a “positive,” and use this as the outcome measure in this study. A person who is eventually recorded experiencing first-time homelessness within our dataset is referred to as a “consequent positive case,” or a “consequent positive.” A “negative case” refers to a person-location-month that is not within twelve months of recorded homelessness and a “consequent negative case” is someone that is never recorded experiencing homelessness in our dataset.

The overall workflow for this study began with data preparation. This task involved deciding what final organization the data required for modeling and then transforming the provided data to that state. Data preprocessing followed, which comprised treating nulls within the dataset, restricting the data range, and engineering new features. The model was then fit to the data and evaluated using the process of Repeated, Stratified K-Folds.

### Data preparation and preprocessing

**Fig 2:**
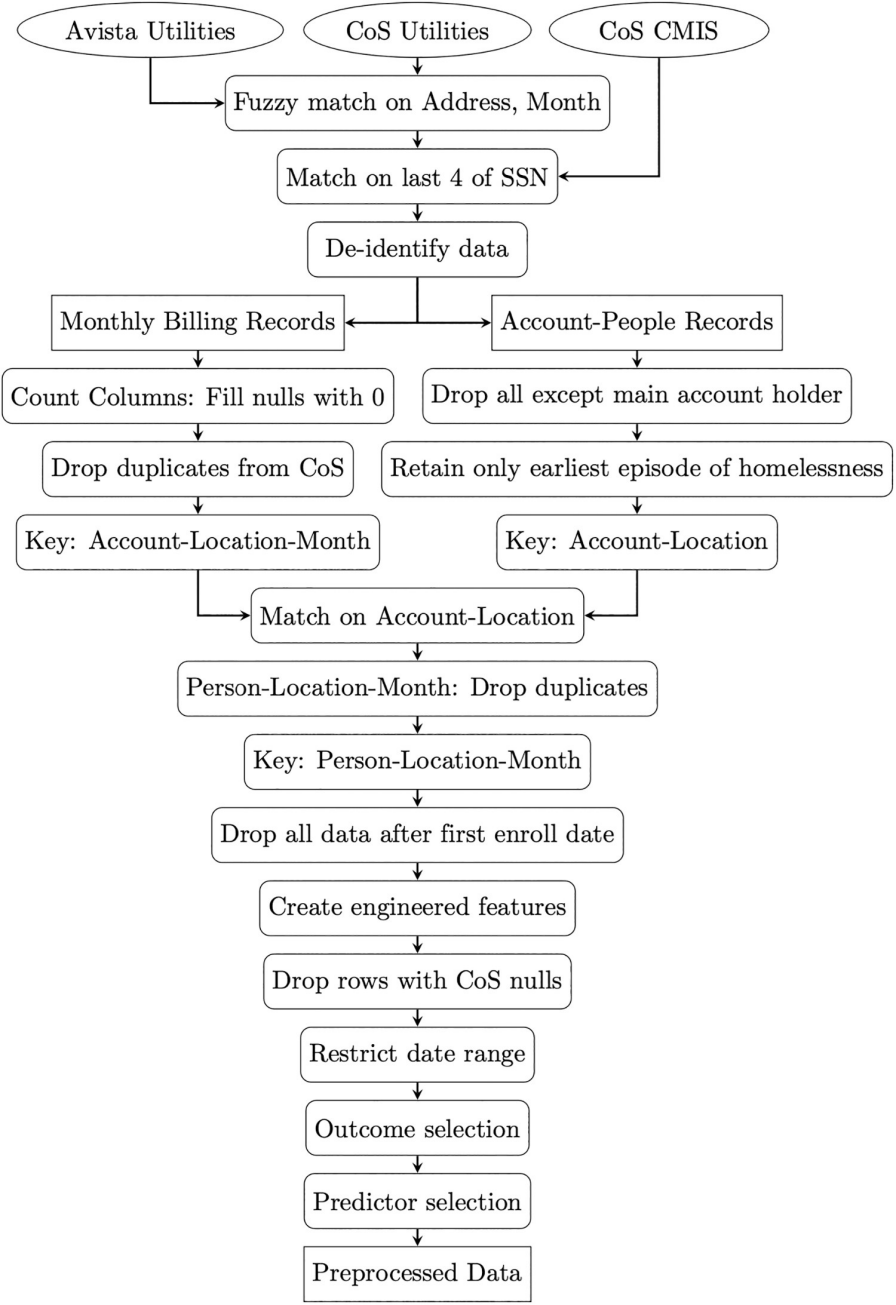
Data preparation and preprocessing diagram. Initial data was provided by Avista Utilities, the City of Spokane Utilities department (CoS Utilities), and the City of Spokane Community Management Information System that tracks homelessness (CoS CMIS). The authors received the data as two file groups, Monthly Billing Records and Account-People Records, after de-identification and initial matching was performed by the Avista data team.

A diagram of data preparation and preprocessing is displayed in Fig 2 that summarizes the steps involved. Initial data matching and de-identifying was performed by the data team at Avista Utilities. Joining utility billing data from Avista and the City of Spokane involved fuzzy matching, an algorithm that pairs entities that are less than a few characters different, on address and month. This dataset was then joined to the City of Spokane’s CMIS homelessness data where matching was based on the last four digits of social security number. After matching, the data was de-identified by Avista Utilities before it was made available to the authors, meaning no individual customer could be identified from the data used in this study. Data de-identification consisted of replacing any identifiable fields such as name, address, and account number with internally generated identification numbers. If this model were put into practice, the predictions from the model would be re-identified by mapping these identification numbers back to the original names and addresses.

After matching and de-identification, the utility billing activity was tied to utility customer accounts, where each account had one or more people associated with it. The billing activity records and account-people records were stored in different tables and required deduplicating before they could be joined. In the account-people table, all accounts had at least one (main) account holder, but many had additional parties associated with them; e.g., co-tenants, landlords, third party family members, and third party agencies. For this study, only the main account holder was retained for each account’s billing activity and all other people associated with the account were removed. This choice was made because the main account holder was the account member ultimately financially responsible for the account and every account had a main account holder. In addition, only the earliest recorded date of experiencing homelessness was retained for each person, ensuring that the model would predict first episode of homelessness. At this point, the account-people data is ready for matching, with composite key of account-location.

The billing records and account-people records were joined on account-location. Some people had multiple billing accounts at the same location and month. This duplication was likely caused when a person changes account types or switches to a new account for some other administrative reason, but does not change address. These duplicates might have been addressed systematically if their representations in the data were consistent, but they were not. This duplication only affected the records of 164 (0.17%) people and all of these were consequent negatives, so all duplicated person-location-months were removed. All billing data occurring after the first recorded episode of homelessness was removed. This ensured the model would predict the first episode of homelessness and not use information about a previous episode of homelessness to predict a subsequent episode. After these steps the data had a composite key of person-location-month (SPA_PER_ID, SPA_PREM_ID, MONTH).

The original data contained billing information from December 2015 to December 2020, but two factors prompted the data be restricted to December 2015—December 2019. The first was to combat right censoring of the data. There is often a time lag between a person’s last utility billing data and when they are recorded as experiencing homelessness, that is, people at the latter end of the data timeframe may experience homelessness but are not yet recorded as homeless. Within the data provided, about 80% of people recorded as experiencing homelessness have a time lag less than one year between their last utility billing data and when they were recorded as experiencing homelessness—see Table 1 for a more complete distribution of time lags. Restricting the right end of the data range reduces the potential for right censoring. The second factor that prompted a restriction of the date range was the COVID-19 pandemic. In 2020 with the onset of the COVID-19 pandemic, behavior of individuals and how utility agencies treated billing changed; for instance, no service shut-offs of utilities were performed by Avista Utilities [18]. The correlations between the predictor variables and the chosen outcome were found to be significantly different in 2020 as shown in S1 Table. Thus, to ensure consistent relationships between the predictor variables and the outcomes, 2020 data use was removed.

**Table 1:** CDF of lag between last billing data and record of homelessness.

Proportion	5%	10%	20%	50%	80%	90%	95%	100%
Lag (Years)	0.011	0.014	0.030	0.082	1.073	1.936	2.238	3.668

The data covers all 303 consequent positives in the original data (December 2015—December 2020). The top row lists the proportion of consequent positives that have a lag, in years, less than the number listed in the second row. For instance, 50% of consequent positives have a lag of 0.082 years (approximately one month) or less between their last recorded utility bill and when they were recorded experiencing homelessness.

After the data preparation steps above, the data covered 90,717 Avista billing accounts, 64,168 locations (premises) in Spokane County, and 86,317 primary account holders which constituted 16.0% of Spokane County residents and 41.8% of households based on the 2020 U.S. Census [19, 20]. The data was monthly and covered from December 2015 through December 2019. Prior to December 2015, Avista used a different account tracking system, and it would have added complexity to combine data from both systems. Only 299 people, or 0.35%, of the people covered in the dataset were consequent positives and only 1,826, or 0.06%, of person-location-months were positives. This extreme imbalance in the dataset is inherent to predicting rare negative socioeconomic outcomes such as homelessness and complicates accurate forecasting of risk of homelessness. See S1 Table for a complete list of all variables and their descriptive statistics after preprocessing and scaling.

In the monthly billing table, any null value in a column containing counting data (BREAK_ARRANGEMENT, BREAK_PAY_PLAN, CALL_OUT, CALL_OUT_MANUAL, DUE_DATE, FINAL_NOTICE, PAST_DUE, SEVERANCE_ELECTRIC, SEVERANCE_GAS, and COVID_REMINDER) was assumed to be 0. This imputation affected 3,126,613 (81.8%) rows of data. There were duplicated data in the city billing records likely from matching issues between Avista and City of Spokane billing records. These duplicates only affected 327 (0.31%) of account-locations in the billing data, so all duplicates were removed.

The next step of data preprocessing addressed missing values in the city data. There are 3,793,093 person-location-months of Avista billing data, but 130,535 (3.44%) of those are missing the corresponding city billing data. These nulls arose either from lack of data coverage on the part of the city utilities or on errors in the matching between city data and Avista data. Two approaches were considered:

1. Drop the person-location-months with no city billing data (Drop Nulls) or
2. Drop the city billing columns altogether (Avista Only).

These options were evaluated by which produced data that was most strongly correlated with the outcome. S2 Table displays the Spearman Correlations between the outcome and each predictor variable for the two options. The correlations for Drop Nulls were slightly higher than those for Avista Only, so the Drop Nulls option was used.

Additional information was extracted from the data by engineering new features. New features were created by aggregating billing information at the following levels: Avista electric, Avista gas, Avista combined, City combined, Avista and City combined. The number of people who have lived at each location was calculated and recorded as NUM_PER_FOR_PREM. This variable was a measure of the “temporariness” of residents at each location and may relate to a location’s attractiveness. The cumulative number of locations where a person has paid utility bills over time was also calculated and recorded as NUM_PREM_FOR_PER. This variable was intended to act as a proxy for the number of places each person has lived, though some people pay utilities at multiple premises for reasons other than moving; they may own multiple properties or pay someone else’s (such as their child’s) utility bills. People may also live in a residence without being listed as the primary utility account holder.

Additional outcome measures were developed and tested. The original data contained only the date at which a person was recorded seeking services based on their homeless status, ENROLL_DATE, and a binary feature for if a person was ever recorded as experiencing homelessness in the CMIS database (CMIS_MATCH). Another outcome was created, MO_AWAY, which recorded the number of months away from homelessness for each person-location-month. Other binary outcomes were created to capture if a person-location-month was within *x* months of experiencing homelessness, where *x* ∈ [1, 3, 6, 12]. The outcome of WITHIN_12_MO, if a person-location-month was within 12 months of experiencing homelessness, was eventually chosen as the model outcome.

### Model

Multivariate binary logistic regression was used to predict if an individual was within 12 months of experiencing first-time homelessness. This type of model has been used for predicting risk of homelessness in several previous studies [8, 13, 15, 21, 22] and is appropriate for prediction of the likelihood of a binary event based on a set of continuous predictors. For each person-location-month’s data fed into the model, the model produces an output of the predicted probability that the person-location-month is within 12 months of experiencing first-time homelessness. Model fitting is performed using maximum likelihood estimation.

We present two models: a model applicable to most households that pay utility bills (“General Model”) and a model specifically applicable to Spokane County (“Spokane Model”). The General Model uses information that is likely available for any household in the developed world that pays utility bills, see Table 4 for a list of the variables included. The Spokane Model includes more information from the local utility agencies to increase model performance, see Table 5 for a list of the variables included.

The numerical outcome of number of months until homelessness, MO_AWAY, was not used because Consequent Negatives, those that are never recorded as experiencing homelessness, have no logical value for MO_AWAY. Of the remaining outcomes, WITHIN_12_MO was chosen because it had the second-highest correlation with predictors to CMIS_MATCH and provided more actionable information. WITHIN_12_MO indicates whether a person will experience homelessness within twelve months while CMIS_MATCH only indicates if a person will ever experience homelessness. S3 Table contains the comparison of correlations between each outcome considered and the predictors.

Different factors were considered for the two models when choosing which predictors to include. Because this modeling task focused on prediction, all predictors were included in the model except those that were highly correlated because these caused problems with model convergence. The process for selecting predictors for the Spokane Model is as follows:

1. Calculate the correlation between each predictor and the outcome.
2. Calculate the correlation between all the predictors.
3. Group predictors that are highly correlated with each other.
4. For each group, retain only the predictor that is most correlated with the outcome.

For the General Model, Step 4 contains the additional requirement that the predictor also be commonly available for most customer-utility situations. The complete list of predictor variables available for model fitting and their definitions is shown in S4 Table.

### Model evaluation

The models were evaluated based on performance accurately predicting the outcome for each person-location-month by comparing the models’ predictions to the actual outcomes. The performance metrics of Area Under the Curve (AUC, more on this below), Accuracy (ACC), True Positive Rate (TPR), False Negative Rate (FNR), False Positive Rate (FPR), True Negative Rate (TNR), Positive Predictive Value (PPV), Balanced Accuracy (BA), and F-1 Score (F1) are all reported for the models. These metrics are based on the model’s Confusion Matrix (explained in Table 2) and the associated formulas can be found in Table 3. Note that in the literature, TPR is also referred to as Sensitivity, Recall, or Hit Rate, while FPR is sometimes referred to as Fall-Out, and PPV is also called Precision in some studies.

**Table 2:** Confusion matrix.

	Predicted Positive	Predicted Negative
Actual Positive	True Positive	False Negative
Actual Negative	False Positive	True Negative

This matrix is used to classify types of predictions. For example, an entity (person-location-month) that is Predicted Positive but is Actually Negative is classified as a False Positive. The numbers of each prediction class enable understanding of the strengths and weaknesses of the model. The reported model metrics described in Table 3 are based on the number of predictions in each prediction class.

**Table 3:** Model performance metrics.

Metric	Formula
ACC	(True Positives + True Negatives) / All Predictions
TPR	True Positives / Actual Positives
FNR	False Negatives / Actual Positives
FPR	False Positives / Actual Negatives
TNR	True Negatives / Actual Negatives
PPV	True Positives / (True Positives + False Positives)
BA	(TPR + TNR) / 2
F1	2 (PPV × TPR) / (PPV + TPR)

The metrics used to evaluate model performance and their associated formulae.

**Fig 3:**
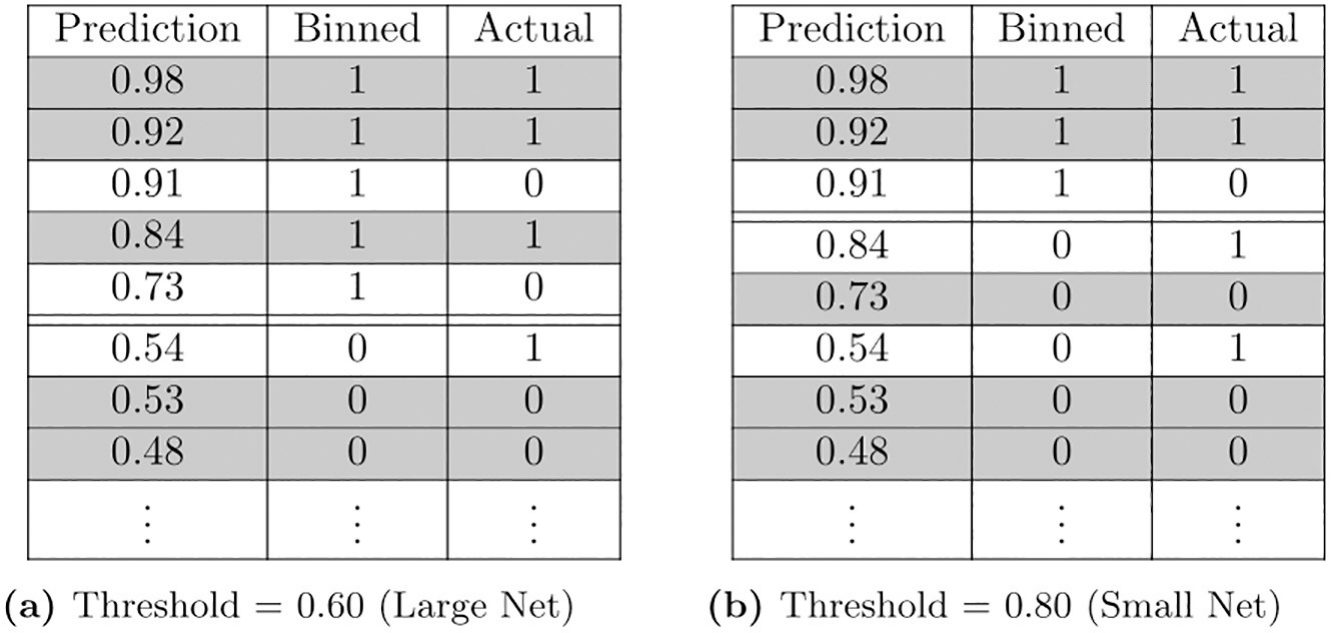
Example of two prediction binning thresholds. The same predictions (Prediction) are binned as a 1 or 0 in the Binned column based on the threshold (represented by the double-horizontal line), then compared to the actual results (Actual) and determined to be correct (gray) or incorrect (no color).

The models were fit to the data and used to predict the outcome of WITHIN_12_MO, but the predictions are likelihoods which are continuous with range from 0 to 1. These must be binned into binary class predictions before they can be compared to the actual binary outcomes. The default binning threshold used here is 0.5, in which case predictions are classified as negative if they are less than 0.5 and positive if they are greater than or equal to 0.5 [23]. However, the binning threshold can be adjusted to better suit model application. The model is intended to identify the people to which an HPP should offer assistance. HPPs that can assist many people could set the model threshold to be relatively low, meaning many predictions are binned as positive, while HPPs that can assist few people could set the threshold relatively high, meaning that only the few, high predicted risks are binned as positive. A demonstrative example of varying the threshold is presented in Fig 3. In practice, setting the binning threshold will likely depend on the resources available to a HPP and the relative costs of False Positives and False Negatives in the context in which the HPP operates.

The most general measure of the model’s performance at all binning thresholds is the AUC, which is based on the Receiver Operating Characteristic (ROC) curve, displayed in the Results section. This provides HPPs with a large amount of information in a concise form. To create this curve, every possible binning threshold is chosen, the binned predictions are evaluated against the actual outcomes, then FPR and TPR are calculated. The FPR and TPR paired values are plotted as points on the ROC curve [24]. The AUC, with range from 0 to 1, is the area under the ROC curve and is a single metric that measures model performance at all thresholds. An AUC of 1 is the theoretical perfect score where the model produces no False Positives and always correctly predicts all Actual Positives at all binning thresholds.

It is standard practice to determine coefficients of a model using a training data set, then evaluate the data on a test data set. This provides an indication of the model’s ability to extrapolate patterns in new data. K-Folds Cross Validation is a model evaluation technique where the data is randomly split into *k* disjoint groups of people, referred to as folds. For each iteration, one fold is treated as the test set and the remaining data acts as the training set. The model is trained and evaluated using this data split, the results are recorded, then the process is repeated using the next fold as the next test set. The purpose of this process is to average model performance across many data splits to mitigate the risk that model performance is based on a non-representative data split. The default number of folds suggested in the literature is ten (*k* = 10), however due to the scarcity of positive cases, we considered five folds (*k* = 5). The rationale is that for data sets with sparse positive events, if the folds are too small, model evaluation is highly sensitive to the few positive cases in the test set [25]. In addition, to mediate relative scarcity off positive events, we utilize Repeated Stratified K-Folds, which differs from the basic K-Folds algorithm in two ways. The first is that the folds are stratified on the outcome, meaning all the folds have similar proportions of positives and negatives to each other and the dataset as a whole. In this case, the data was randomly stratified on people so that the proportion of people who will eventually experience homelessness was similar across splits. This technique allows the model to train and test on data that is as representative as possible. The second difference is that the entire process of creating folds, splitting data, fitting a model, and evaluating the model is repeated *n* times, where here we set *n* = 10. This repetition functions as additional assurance that the splitting has not happened to occur in such a way that allows the model to perform either unrealistically well or poorly [26]. Technically there is a different model (i.e., coefficients resulting from the logistic regression fitting routine) for each of the folds, so in our case there are *k* * *n* = 5 × 10 = 50 models generated. Model evaluation metrics are calculated for each model derived from the training set. The mean and standard deviation of the metrics are calculated over all models. Repeated Stratified K-Folds is used as a technique to ensure that the reported model performance is representative and repeatable. Listed parameter estimates for the General and Spokane Models are found by fitting these two models on the entire dataset without the use of Repeated Stratified K-Folds.

In order to improve model convergence, the method of *z*-score standardization was performed for each fold. After the data split was performed for each fold, the data was scaled using Eq 1. The sample mean and standard deviation, *u* and *s*, of each feature are found using the training set of each fold, then the scaling process is applied to both the training and test sets of each fold. The impact of this type of scaling is that each feature value becomes a measure of the distance, in sample standard deviations, the value is from the feature mean. The transformed features allow model training to be performed much faster and allow the coefficients to be compared.

$$\begin{array}{c} z_{entity,feature}=\frac{x_{entity,feature}-u_{feature}}{s_{feature}} \end{array}$$ (1)

To combat the high degree of class imbalance in the dataset, the Synthetic Minority Oversampling Technique (SMOTE) was employed. SMOTE generates synthetic examples of the minority (positive) class that are similar to real examples. This process effectively balances the dataset so that the model will understand the importance of correctly predicting the positive cases [27]. This oversampling technique is applied only on the training set, not the testing set. The complete list of steps for model evaluation are as follows:

- Randomly split the data into *k* folds and *n* repeats based on person and outcome so that similar ratios of positives to negatives appear in each fold.
- For each configuration of folds (training and testing sets),
  - Use SMOTE to balance the data in the training set.
  - Scale the predictor variables in the training set using *z*-score standardization.
  - Fit a logistic regression model to the data.
  - Use the same scaling parameters to scale the predictor variables in the testing set.
  - Use the model to predict the outcomes for the testing set.
  - Evaluate the model based on these predictions.
- Take the mean and standard deviation of the performance metrics across all folds.

## Results

**Fig 4:**
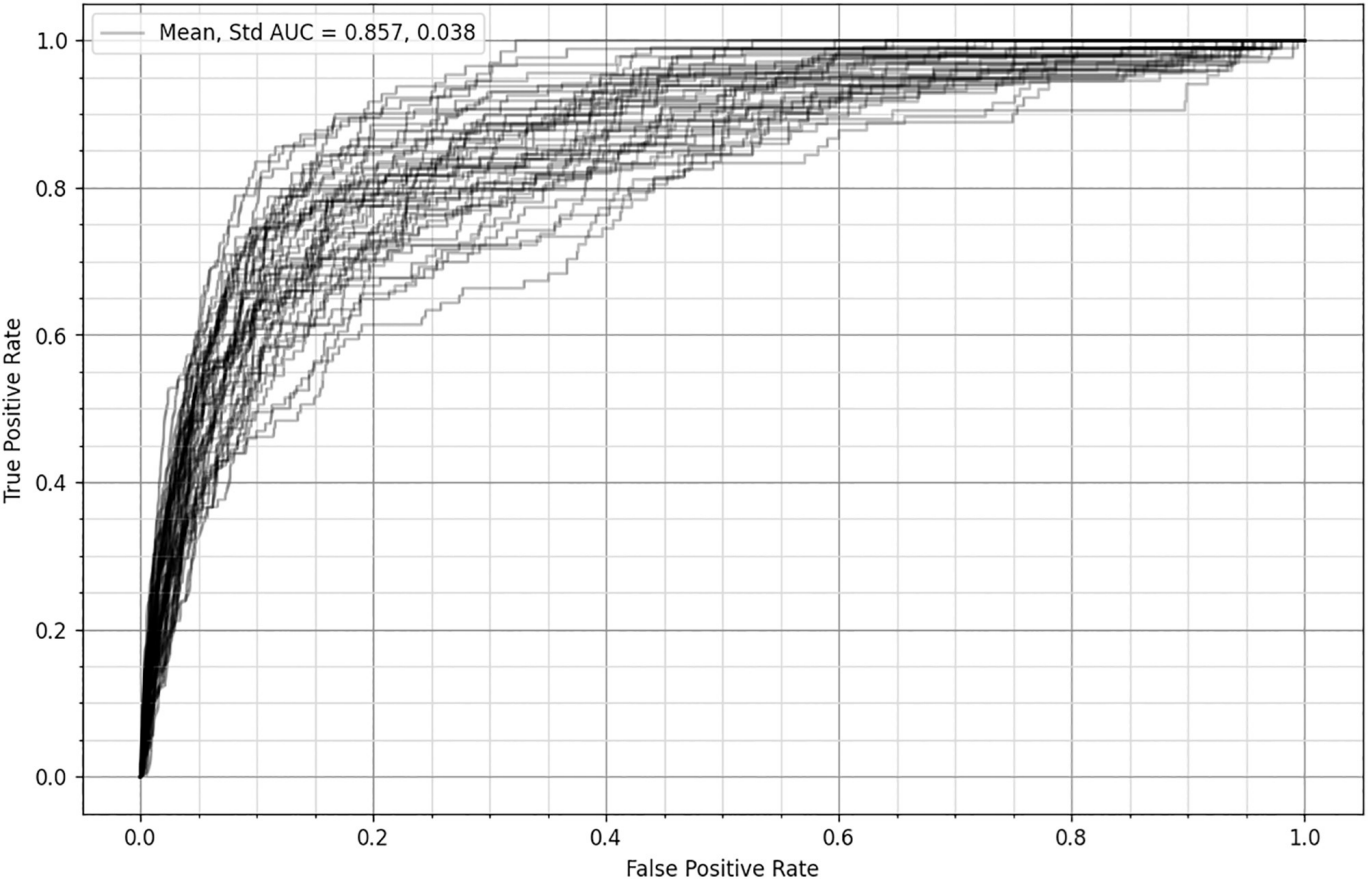
The Receiver Operator Characteristic curve for the General Model. This plot was created by choosing every possible binning threshold, then at each threshold evaluating FPR and TPR of the General Model. The plot consists of only the (FPR, TPR) points. FPR (False Positive Rate) is the proportion of False Positives to the True Negatives. TPR (True Positive Rate) is the proportion of Predicted Positives to the True Positives. A model that performs perfectly would have an FPR of 0 and a TPR of 1. AUC (Area Under the Curve) is 0.857, measured against the theoretical perfect score of 1.000.

In order for the model to be applied in practice, a specific binning threshold must be chosen with different thresholds yielding different model performances. The appropriate threshold will depend upon the relative costs of False Positives to False Negatives in the specific context the model is applied. For example, if a HPP uses an expensive intervention, the cost of False Positives can be large, so a high threshold with fewer False Positives is preferred, but if an inexpensive intervention is used in the same context the cost of False Positives is smaller so a lower threshold that correctly identifies more positives (but also misclassifies more True Negatives as positive) is preferred. High thresholds correspond to the lower left region of the ROC plot (Fig 4) where the TPR and the FPR are both low because few people are predicted as positive.

The coefficient for each feature, shown in Table 4 for the General model and Table 5 for the Spokane Model, signifies the weight of importance of each feature to make a prediction. For each feature, if the value were to increase by one standard deviation with all other features held constant, the coefficient represents the resulting change in predicted log-odds of the outcome. A positive coefficient means that a feature value above the feature mean is predictive of the outcome. A negative coefficient conveys that a feature value above the feature mean is protective against the outcome. All the coefficients for the General Model are significant at the 95% confidence level (p-value < 0.05) as well as 21 of the 24 coefficients for the Spokane Model.

**Table 4:** General Model coefficients.

Variable	Coef	Lower	Upper	p-value
NUM_PER_FOR_PREM	0.5374	0.5356	0.5391	< 0.001
TOTAL_CUR_BAL_AMT	0.4071	0.4046	0.4096	< 0.001
PAST_DUE	0.3773	0.3753	0.3792	< 0.001
BREAK_ARRANGEMENT	-0.0089	-0.0106	-0.0073	< 0.001
NUM_PREM_FOR_PER	-0.1689	-0.1718	-0.1660	< 0.001

Model coefficients for the General Model fit to all the data. Lower and Upper list the lower and upper bounds for the 95% confidence interval of each coefficient, respectively. For PAST_DUE the coefficient can be interpreted as, “If PAST_DUE is increased by one standard deviation with all other parameters held constant, then the log-odds of the outcome are increased by 0.3773.”

**Table 5:** Spokane Model coefficients.

Variable	Coef	Lower	Upper	p-value
NUM_PER_FOR_PREM	0.5522	0.5503	0.5540	< 0.001
RES_EL_CUR_BAL_AMT	0.4779	0.4478	0.5079	< 0.001
FINAL_NOTICE	0.3140	0.3119	0.3161	< 0.001
CITY_90_DAYS_PAST_DUE_AMT	0.1708	0.1637	0.1779	< 0.001
CITY_30_DAYS_PAST_DUE_AMT	0.1436	0.1404	0.1467	< 0.001
RES_GAS_OVER_120_DAYS	0.1338	0.1149	0.1527	< 0.001
CITY_60_DAYS_PAST_DUE_AMT	0.0818	0.0795	0.0841	< 0.001
RES_GAS_CUR60_DAYS	0.0528	0.0423	0.0632	< 0.001
RES_GAS_CUR30_DAYS	0.0421	0.0328	0.0514	< 0.001
RES_GAS_CUR120_DAYS	0.0243	0.0208	0.0279	< 0.001
RES_GAS_CUR90_DAYS	0.0220	0.0165	0.0275	< 0.001
RES_EL_CUR120_DAYS	0.0005	-0.0028	0.0038	0.906
RES_GAS_CUR22_DAYS	-0.0039	-0.0277	0.0198	0.102
RES_EL_CUR30_DAYS	-0.0054	-0.0134	0.0025	0.543
CALL_OUT_MANUAL	-0.0093	-0.0129	-0.0057	< 0.001
RES_EL_CUR90_DAYS	-0.0161	-0.0213	-0.0110	< 0.001
BREAK_PAY_PLAN	-0.0190	-0.0207	-0.0173	< 0.001
RES_EL_OVER_120_DAYS	-0.0260	-0.0295	-0.0225	< 0.001
SEVERANCE_GAS	-0.0297	-0.0334	-0.0260	< 0.001
CITY_TOT_DUE	-0.0591	-0.0661	-0.0522	< 0.001
RES_EL_CUR60_DAYS	-0.0758	-0.0853	-0.0663	< 0.001
RES_GAS_CUR_BAL_AMT	-0.0873	-0.1253	-0.0494	< 0.001
NUM_PREM_FOR_PER	-0.1724	-0.1754	-0.1694	< 0.001
RES_EL_CUR22_DAYS	-0.1774	-0.2005	-0.1542	< 0.001

Model coefficients for the Spokane Model fit to all the data. Lower and Upper list the lower and upper bounds for the 95% confidence interval of each coefficient, respectively. For FINAL_NOTICE the coefficient can be interpreted as, “If FINAL_NOTICE is increased by one standard deviation with all other parameters held constant, then the log-odds of the outcome are increased by 0.3140.”

For the General Model, BREAK_ARRANGEMENT and NUM_PREM_FOR_PER were found to be protective against homelessness due to their negative model coefficients, while NUM_PER_FOR_PREM, TOTAL_CUR_BAL_AMT, and PAST_DUE were found to be associated with increased risk of homelessness. It is surprising that BREAK_ARRANGEMENT is protective since that is the number of times a service shutoff arrangement was established for a person. Perhaps a customer’s willingness and ability to enter an agreement with the utility company is an indicator of the customer’s level of responsibility. Similarly, it is surprising that NUM_PREM_FOR_PER is protective since this means that if a person has been the main utility account holder at more locations, or premises, the model predicts them to be at a lower risk of homelessness. This contradicts the hypothesis that people at risk of homelessness are more transient than the general population. Seemingly contradictory relationships appear in the coefficients of the Spokane Model—see Table 5. For example, RES_EL_CUR22_DAYS is the largest negative coefficient while RES_EL_CUR_BAL_AMT has the second-largest positive coefficient. Several features have coefficients that are opposite the sign expected, such as the protective features SEVERANCE_GAS and BREAK_PAY_PLAN. These results are likely an indication that the Spokane Model has over-fit the intercorrelated data. However, the features selected for the General Model were not intercorrelated and so the coefficients are meaningful at an association level.

The values for many useful performance metrics are listed in Table 6. Including this nearly exhaustive list allows for easy comparison between our research and other work. Perhaps the most important metric in this context is the True Positive Rate (TPR). This metric measures the proportion of the Actual Positives that were Predicted Positive by the model. Only about 0.06% of the data are Actually Positive cases, but the General Model predicted 34.72% of cases as positive and the Spokane Model predicted 35.12% of cases as positive. Most of the positive predictions for both models are False Positives, but the General Model captures 86.2% of Actual Positives and the Spokane Model captures 87.7%. These calculations were performed at the default prediction binning threshold of 0.5, however, HPPs can tailor the model to fit their needs by adjusting the threshold. These metrics are sourced from the model confusion matrix shown in Table 7 which provides deeper insight into what predictions each model is making.

**Table 6:** Model performance.

	General Model		Spokane Model	
	Mean	Std	Mean	Std
AUC	0.857	0.038	0.860	0.036
ACC	0.653	0.018	0.649	0.024
TPR	0.862	0.064	0.877	0.061
FNR	0.138	0.064	0.123	0.061
FPR	0.347	0.018	0.351	0.024
TNR	0.653	0.018	0.649	0.024
PPV	0.002	0.000	0.002	0.000
BA	0.757	0.032	0.763	0.031
F1	0.003	0.000	0.003	0.000

The mean and standard deviation of each performance metric across *k* folds and *n* repeats. Metrics reported for both the General and Spokane Models.

**Table 7:** Model confusion matrix.

	General Model		Spokane Model		Total
	Predicted P	Predicted N	Predicted P	Predicted N	
Actual P	1,603	242	1,634	212	1,845
	(0.053%)	(0.008%)	(0.054%)	(0.007%)	(0.06%)
Actual N	1,048,740	1,974,771	1,060,720	1,962,760	3,023,511
	(34.665%)	(65.274%)	(35.061%)	(64.877%)	(99.94%)
Total	1,050,343	1,975,013	1,062,354	1,962,972	3,025,356
	(34.72%)	(65.28%)	(35.12%)	(64.88%)	(100.00%)

The mean number (proportion) of each type of model prediction over across the *k* folds and *n* repeats. P and N stand for Positive and Negative, respectively.

## Discussion

The ubiquity, availability, and timeliness of utility payment history has potential for use with HPPs to identify those in the general population potentially at risk of experiencing homelessness. The models developed in this work could effectively act as an initial screening tool in a multistep screening system. Both the General and Spokane Models effectively predict the Actual Positive cases (achieved TPRs of 0.862 and 0.877, respectively), however this high performance comes at the cost of predicting many False Positives. The General and Spokane Models produce, on average across the *k* folds and *n* repeats, 34.72% and 35.12% Predicted Positives, respectively, while the proportion of Actual Positives is only about 0.06%. Thus, use of these models is warranted when the cost of a False Negative is much higher than the cost of a False Positive. Here False Negatives correspond with a model failing to identify an individual that will experience homelessness, while a False Positive corresponds to classifying someone as at risk when the person does not experience homelessness. However, presumably some of these individuals identified as at risk may be maintaining precarious financial insecurity and could benefit from assistance.

As resources to assistance programs are finite, additional screening could be employed to identify those most at risk based on other data. The initial step would be to identify people at the population level that are more likely at risk of experiencing homelessness, then the HPP could reach out to these individuals using some low-cost method to request further information about their financial situation or invite them to apply for assistance. After further information is gathered, additional screening steps may be applied. If a HPP wishes to implement a higher-cost intervention, these models could be tuned to produce fewer Predicted Positives by increasing the binning threshold. In this scenario, the PPV is increased at the cost of reducing TPR.

Our models have different strengths than those in Byrne et al. [15]. Table 8 displays a comparison of the shared performance metrics of our models with the model in [15], which utilized healthcare and criminal history data. Notably, the Byrne et al. model achieves significantly better PPV and AUC metrics, indicating their model is more accurate in its positive predictions and generally had better performance in TPR and FPR over all binning thresholds. However, our models achieved significantly better TPRs which is the most important metric in this context. These metrics indicate that Byrne et al.’s model was more precise, but our models correctly predicted a greater portion of the Actual Positives. The data sources in [15] are highly relevant to the outcome of homelessness as evidenced by their high model performance, but the timeliness of the data, and therefore the ability for the model to be productionalized, is limited [15].

**Table 8:** Comparison of model performance.

	General Model	Spokane Model	Byrne et al. Model
AUC	0.857	0.860	0.940
TPR	0.862	0.877	0.778
TNR	0.653	0.649	0.951
PPV	0.002	0.002	0.117
BA	0.757	0.763	0.864

Comparison of model performance between the presented General and Spokane models with the model of Byrne et al. [15]. The models in this work utilized utility payment history, while healthcare and criminal history were used as predictors in [15].

We present our models as initial screening tools, however, combining utility customer billing data with other sources, such as healthcare or criminal history, has the potential for a more comprehensive statistical model that would almost certainly result in improved performance. The main obstacle to this route is the timeliness and accessibility of these additional data sources. There are two main limitations to the utility billing data used for this project: lack of coverage and uncertainty in negative labels. This data source was chosen because it was conjectured to contain financial health information for members of the general population, but because account histories only provide information on the main account holder, only about 16.0% of the total Spokane County population was represented in the data [19]. Not considered in this work, but future study is warranted on the risk of the associated co-tenants or members of a household listed in the account information. For instance, if the main account holder is identified as at risk of homelessness, a natural assumption may be that the co-tenants are also at increased risk of experiencing homelessness. Another limitation to the data was uncertainty in the negative outcome labels. Positive labels have a high confidence in correctness—people labeled as positive almost certainly did seek assistance based on their condition of homelessness. Some people labeled as negative, though, may have experienced homelessness but were simply never recorded. This unquantified factor in the data may have negatively impacted model performance. A common approach to combat this limitation is to use a proxy outcome measure. This approach should be investigated in future work after adequate study of appropriate proxies.

In spite of these limitations, utility payment history remains an attractive source of information for predicting homelessness. Because of its ubiquity, availability, timeliness, and relevance to homelessness, utility payment history has great potential for use with HPPs to identify those in the general population at risk of experiencing homelessness.

## Conclusion

By creating a predictive model of homelessness based on residential utility customer billing information that scores highly in important performance metrics, we have demonstrated the usefulness of this data for the task of predicting homelessness. While this data does not cover the entire population, it does reach a large proportion of households and is likely widely available in most capitalist economies. It is also updated regularly, usually monthly, and would enable predictions at the same cadence. This finding lays the foundation for future work to extract additional information from these data, combine the data with other relevant sources, and build more sophisticated models.

The models presented in this paper have performance similar to the current research while predicting on people not already seeking assistance. This allows either of these models to act as an initial screening tool to identify people that previous methods would have missed. Using these models, homelessness prevention programs would have the ability to reach out to people predicted to be at high risk of experiencing homelessness, lowering the barriers to providing preventative assistance to people in need.

## Supporting information

S1 Fig Spearman Correlation of the correlation of each predictor with WITHIN_12_MO by year. For each year, the correlation between each predictor and WITHIN_12_MO was determined, then the correlation of these values between years was calculated. This was used to determine if relationships between predictors and outcome remain consistent over time. Since the year 2020 is weakly correlated with the other years, it likely contains data relationships that differ from other years.
(TIFF)

S1 Table Predictor descriptive statistics. Descriptive statistics for the untransformed features. Note that after scaling, a feature value of 1.0 means one feature standard deviation above the feature mean.
(TIFF)

S2 Table Spearman correlation between predictors and WITHIN_12_MO for data containing city columns (Drop Nulls) and without (Avista Only). The Drop Nulls data excludes people-location-months with no City billing data. The Avista Only data excludes all the city billing data. The Drop Nulls configuration had higher correlation of the predictors with the outcome so that was used instead of Avista Only.
(TIFF)

S3 Table Spearman Correlation of each predictor for several outcomes. This table was used to help determine which outcome to use. Here CMIS refers to the outcome CMIS_MATCH, 1_MO to 1_MO_AWAY, etc. The names were shortened for chart formatting.
(TIFF)

S4 Table Complete list of variables available and their descriptions. (TIFF)
