# Supplementary figures and images for: The value of utility payment history in predicting first-time homelessness
Source: PLoS One. 2023 Oct 9;18(10):e0292305. doi: 10.1371/journal.pone.0292305 (PMC10561862; doi:10.1371/journal.pone.0292305)

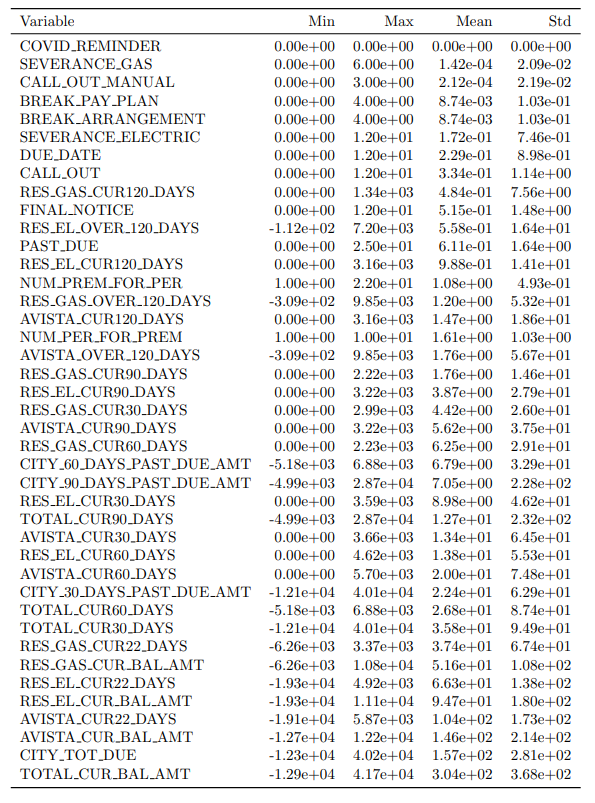

Supplement: S1 Table — Descriptive statistics for the untransformed features. Note that after scaling, a feature value of 1.0 means one feature standard deviation above the feature mean. (TIFF) [file pone.0292305.s002.tiff]

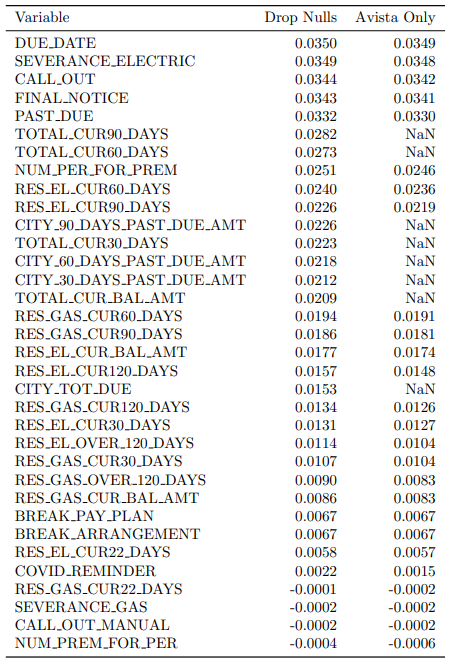

Supplement: S2 Table — The Drop Nulls data excludes people-location-months with no City billing data. The Avista Only data excludes all the city billing data. The Drop Nulls configuration had higher correlation of the predictors with the outcome so that was used instead of Avista Only. (TIFF) [file pone.0292305.s003.tiff]

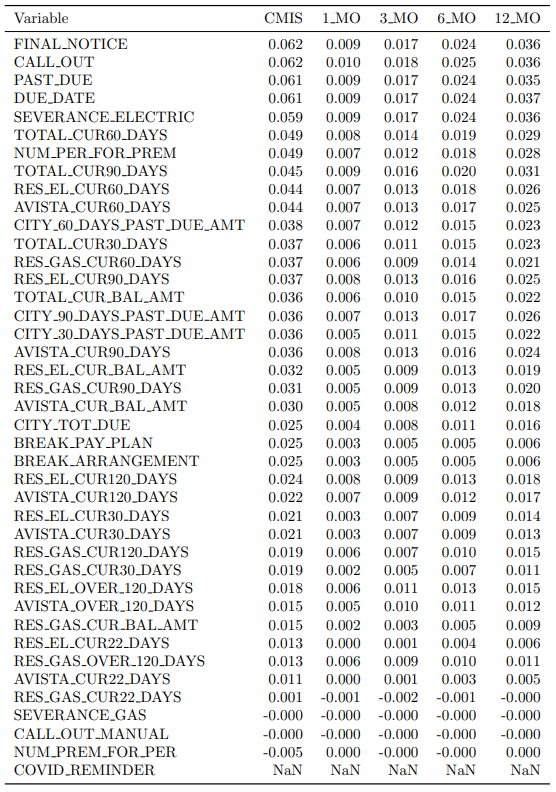

Supplement: S3 Table — This table was used to help determine which outcome to use. Here CMIS refers to the outcome CMIS_MATCH, 1_MO to 1_MO_AWAY, etc. The names were shortened for chart formatting. (TIFF) [file pone.0292305.s004.tiff]

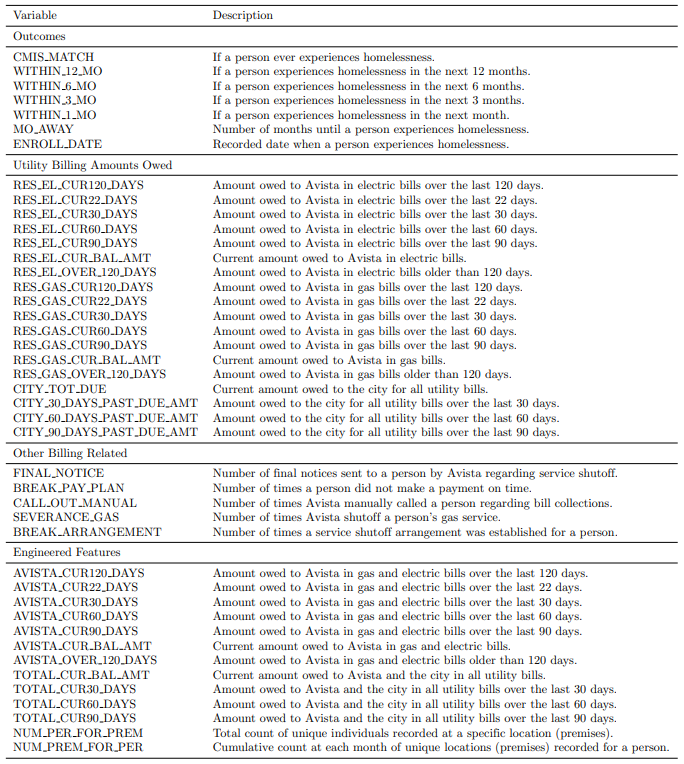

Supplement: S4 Table — (TIFF) [file pone.0292305.s005.tiff]
